# Supplementary material for: The Effectiveness of Massage Therapy for Improving Sequelae in Post-Stroke Survivors. A Systematic Review and Meta-Analysis
Source: Int J Environ Res Public Health. 2021 Apr 21;18(9):4424. doi: 10.3390/ijerph18094424 (PMC8122530; doi:10.3390/ijerph18094424)
Supplement: Supplementary file 1 [file ijerph-18-04424-s001.zip › ijerph-1131854-supplementary.pdf]

Supplemental Table S1: PEDro scale.

Author, year	Item 1	Item 2	Item 3	Item 4	Item 5	Item 6	Item 7	Item 8	Item 9	Item 10	Item 11	score
Mok, 2004	YES	YES	NO	YES	NO	NO	NO	YES	NO	YES	YES	5
Jin-su, 2005	YES	YES	NO	YES	NO	NO	NO	NO	NO	YES	YES	4
Amanullah 2011	YES	YES	NO	YES	YES	NO	NO	YES	YES	YES	YES	7
Pan , 2011	YES	YES	YES	YES	NO	NO	YES	YES	YES	YES	YES	8
Yang , 2011	YES	YES	NO	YES	NO	NO	NO	YES	YES	YES	YES	6
Zarnigar, 2012	YES	YES	NO	YES	NO	NO	NO	YES	NO	YES	YES	5
Li, 2012	YES	YES	NO	YES	NO	NO	NO	YES	YES	YES	YES	6
Wu, 2013	YES	YES	NO	YES	NO	NO	NO	YES	YES	YES	YES	6
Zhang X, 2013	YES	YES	NO	YES	NO	NO	NO	YES	YES	YES	YES	6
Zhang Y, 2013	YES	YES	NO	YES	NO	NO	NO	YES	NO	YES	YES	5
Thanakiatpinyo 2014	YES	YES	YES	YES	NO	NO	YES	YES	YES	YES	YES	8
Ahmed, 2015	YES	YES	NO	YES	NO	NO	NO	YES	YES	YES	YES	6
Han, 2015	YES	YES	NO	YES	NO	NO	NO	YES	YES	YES	YES	6
Di, 2017	YES	YES	NO	YES	NO	NO	NO	YES	YES	YES	YES	6
Lee, 2017	YES	YES	NO	YES	NO	NO	NO	YES	YES	YES	YES	6
Yang, 2017	YES	YES	YES	YES	YES	NO	YES	YES	YES	YES	YES	9
Wang, 2019	YES	YES	YES	YES	NO	NO	YES	YES	YES	YES	YES	8
Xie, 2019	YES	YES	NO	YES	NO	NO	NO	YES	YES	YES	YES	6

Item 1: Eligibility criteria **(**this criterion influences external validity but not the internal validity of the trial and it is not used to calculate PEDro score), item 2: random allocation, item 3: concealed allocation, item 4: baseline comparability, item 5: blind subjects, item 6: blind therapists, item 7: blind assessors; item 8: adequate follow-up (less than 15% drop-outs),item 9: intention-to treat analysis, item 10: **b**etween groups comparisons, item 11: point estimates and variability

Supplemental Table S2: Parameters and description of interventions

Author, year	Number of sessions		Aplication point	Discription intervention EG and CG
	EG	CG		
Mok, 2004	10' slow-stroke back massage at night for 7 days	NR	Afected shoulder and spine (skull-sacrum)	**EG:** Firmly grasp the top of the patient's shoulders with both hands and place the thumbs of each hand just below the base of the skull, making tiny circular movements on the upper neck. Place the palm of one hand at the base of the skull and make a long, smooth stroke all the way down the patient's spine to his/her waist. The 2º hand follows the first at the base of the skull and strokes down the spine as the first hand returns to the base of the skull, to activate all the points situated along the spine which mobilizes energy and blood to all the organs and guts. Place your hands on either side of the neck under the patient's ears and stroke down and over the patient's collarbones with your thumbs just over the shoulder blades. Repeat the motion several times. Place the thumb of each of your hands beside the spine, beginning with the shoulders, and move the thumbs down the spine to the waist. Repeat several times. Finish by placing your palms on each side of the patient's neck and make continuous, long sweeping strokes down the neck, across each shoulder, and down the back near the spine. Repeat the entire pattern several times. Massatge weas applied by nurses.<br>**CG**: NR
Jin-su, 2005	50' Tuina massage + 25' Acupuncture treatment duration NR	Western medicine (drugs) treatment duration NR	Paretic upper/lower limbs	**EG: Acupuncture**: Baihui (GV 20), Jianyu (LI 15), Quchi (LI 11), Shousanli (LI 10), Waiguan (TE 5), I-Iegu (LI 4), Zusanli (ST 36), Yanglingquan (GB 34), Sanyinjiao (SP 6), and Taichong (LR 3). In subacute phase 4 weeks after the onset acupoints Shuigou (GV 26), and Jiquan (HT 1), Neiguan (PC 6), Huantiao (GB 30) and Weizhong (BL 40) in the affected side were punctured with twirling, lifting and thrusting techniques until the needled limbs twitched for 3 times, removing the needles immediately. Later, Motor Area and Sensory Area on scalp were needled, and Bafeng (Ex-LE 10) and Baxie (Ex-UE 10) were pricked with needle to bleed slightly in the skin. Reinforcing qi and dredge collaterals. Warming needle method, using moxa in the top of the needle increasing acupuncture effect, was applied on Zhongwan (CV 12), Tianshu (ST 25), Qihai (CV 6), Zusanli (ST 36), and Quchi (LI 11) to strengthen Yuan-qi for 25 rain. Bleeding technique is used to activate the blood flow to the activated point and to detoxify the blood.<br>**EG: Tuina Massage:** Knead Yintang (Ex-HN 3), Jingming (BL 1), Taiyang (Ex-HN 5), Jiaosun (TE 20), Fengchi (GB 20), Fengfu (GV 16), Jianjing (GB 21), Motor Area, and Sensory Area, press Fengfu (GV 16), lastly grasp Fengchi (GB 20) and Jianjing (GB 21).Roll the affected limbs; knead Jianyu (LI 15), Quchi (LI 11), Waiguan (TE 5), Hegu (LI 4), Fengshi (GB 31), Zusanli (ST 36), Yanglingquan (GB 34), Xuanzhong (GB 39), and Kunlun (BL 60); grasp, pluck and knead the upper and lower limbs, and lastly move hip, knee, ankle, shoulder, elbow, and wrist joints.<br>**CG:** Drugs to lower intracranial pressure and maintain the blood pressure, according to the patient's condition, drugs for improving brain blood circulation and supply nutrition to the brain cells.
Yan, 2007	30'Tuina massage (Ju-ci) for 60 days	30'Tuina massage for 60 days	All the body	**EG: Basic maneuvers:** Head and face: Place the patient in the supine position, first push the Yintang towards the Shenting and then push the Yintang along the eyebrows up to the eyebrows, back and forth 8-10 times. Touch the Touwei, Baihui and Fengchi acupuncture points for 3' to 5', and then rub your face for 3'. Finally, apply the five finger clamping method on the head 3-5 times. Upper limb operation: the patient is in supine or lateral decubitus, using the kneading methods and the gun technique for 8-10' on the joints of the shoulders and upper extremities, and then press and rub the shoulders, arms, Quchi, Shousanli, Hegu,until redness and mild discomfort appear. Cooperates with passive flexion, extension, and rotation of the upper limbs. Lower back and lower limbs: the patient is in the prone position, first press the spine, press 2 sides of the spine and the back of the lower extremities, press the Dumai and Jing of bladder 5- 8 times, focus on Ganshu, Shenshu, and the lower extremities Huantiao, Chengfu, Yinmen, Weizhong, Chengshan, etc. acupuncture points, until a desired sensation (De Qi) comes out; The patient in supine position first relaxes with the gun technique for the lateral, anterior and medial side of the lower extremities, then press and rub the points such as Tiguan, Fengshi, Futu, Xuehai, Liangqiu, Kneeyan, Zusanli, Sanyinjiao and Xiexi, until redness and mild discomfort appear; Cooperates with passive flexion, extension, and rotation of the lower extremities.<br>**Tuina massage (Ju-ci):** First performed a pulse diagnosis on both sides of the patient's cunkou to identify the pulse magnitude, deficiency, excess, cold and heat of the condition, combined with the strength of the patient's physique, to determine the application of the method Ju-Ci. Ju-Ci method application: If both sides have strong pulses, and the patient has a normal body constitution, the treatment is mainly on the healthy side, and the tuina technique is used to alleviate the effect. If both sides have weak pulses and the

				body constitution is weak, the treatment is mainly on the healthy side, and the tuina technique is used to compensate for the effect. If one side of the pulse is strong and the other side of the pulse is weak, treatment is applied to both affected sides at the same.<br>**CG:** The treatments on the head, face and lower back were the same as in the EG. The upper and lower limbs are mainly treated on the affected side. The tuina massage technique is adapted to individual conditions and the strength of the treatment is based on the differentiation of the syndrome.
Amanullah, 2011	10′ Massage with *Roghan Seer* alternative days for 45 days	10′ Sham Massage with petroleum jelly alternative days for 45 days	Paretic upper and lower limbs and back	**EG:** Firm pressure and stroking with hands and *Roghan Seer* Unani medicine<br>**CG:** Firm pressure and stroking with hands and petroleum jelly Unani medicine
Pan HP, 2011	40′ Tuina massage 2/day per 5 days/week for 8 weeks 80 sesions	40′ CP 2 times/day per 5 days/week for 8 weeks	Paretic upper and lower limbs and back	**EG:** Unblocking meridians and Du-channel massage Tuina, gun or balance, kouji o percussion techniques on Dumai and bladder and lumbar spine extension. Finger pressure on Jiaji and bladder chanels (along the spine, down the back of legs, finishing in the feet) Dazhui, Shenshu, Dachangshu, traditional most-known acupuncture points: mingmen, Huantiao, Yinmen, Chengfu, Weizhong, Chengshan on lower limb Juyi, Fengshi y Yanglingquan balancing method along the YangMing channel (stomach channel). Yangming and knee flexion and traction and acupresion on Futu, Liangqiu, Kneeyan, Zusanli, Qiuxu, Xiexi, Taichong , Na Weizhong, Chengshan, Kunlun y balancing technique in Jianging (GB 21. Gallbladder channel, in the middle of superior fibers of supraespinatus), from shoulder to metacarpophalangeal joints.Passive mobilitzations on shoulder flexion and abduction, acupresion in Jian Neiling, Quchi y Hegu,<br>**CG**: CP: NDT trunk control and sitting balance exercies, gait between paralel bars and training of activities of daily living
Yang, 2011	50′Massage therapy + CP 1/day per 45 days	50′CP 1/day per 45 days	Paretic upper limb	**EG**: **Tuina massage**: The patient is placed in a supine position, and the therapist applies finger massage to the Jianzhen, Jianfu, Quchi, Shousanli, Yanggu, Yangchi, Yangxi and Hegu points, and applies palm massage to the pectoralis major and trapezius muscles, Subspinous muscles , subscapularis, deltoids, biceps, triceps and brachioradialis around the scapula and upper arm; then use the five finger pinch method, alternately squeeze the two hands from the distal end of the limb to the proximal end10-15 times, then move around the wrist and elbow joints, then rub the affected limb and rub the palm and fingers of the patient; Finally, gently touch the affected limb to finish the treatment. The massage technique during the treatment process should be gentle and delicate, so as not to cause pain.<br>**CP**: Scapula control training: Passive movement of the scapula.Repeat10 actions per group, 1-2 groups each time. Tightening the joints of the chest wall of the scapula. The patient is placed supine with the therapist on the right side of the patient. Grasp the patient's scapula with the left palm and push it upward, while the right hand supports the pectoralis major on the affected side. Squeeze slowly and maintain force for more than 15′, until the tension of the spasm muscle around the shoulder joint decreases. Actively move the scapula: Repeat 10 actions per group, 1-2 groups each time. Scapular Muscle Resistance Training in supine position, the above 2 actions are 10 per group, 1-2 groups each time.
Tong, 2012	Tuina massage + 40′ acupuncture + CP 1/day per 6/week for 4 weeks 24 sessions	Tuina massage + CP 1/day per 6/week for 4 weeks	Paretic upper limb and scalp	**EG**: **Tuina massage**: Digital Anpress and rou knead (drawing circles while pressing the point) (TE 14), Jianzhen (SI 9), Naoshu (SI 10), Jianyu (LI 15), Quchi (LI 11), Yanggu (SI 5), Zusanli (ST 36), Yangchi (TE 4), Yangxi (LI 5), Hegu (LI 4). Rou-knead the pectoralis major muscle, trapezius, deltoid, infraspinatus, subscapularis, biceps brachii, triceps brachii, brachioradialis muscle periscapular and upper arm muscles with palm. Squeeze the limbs alternatively by two hands for 10 to15 times from distal to proximal by 5 fingers Nie-pinching method. Switch the wrist and elbow, Cuo-rub the affected limb, Nian-twist and Rou-knead the palm and fingers. Pai-tap the affected limb gently. Tuina manipulation should be gentle and painless.<br>**Acupuncture**: Major acupoints: Baihui (GV 20), Jianyu (LI 15), Binao (LI 14), Jiquan (HT 1), Jianqian (Extra), Jianhou (Extra), Hegu (LI 4). Adjunct acupoints: Jianjing (GB 21), Tianzong (SI 11), Quchi (LI 11). Point-through-point needling method was used for the major acupoints, such as puncturing from Baihui (GV 20) to Taiyang (Ex-HN 5), from Jianyu (LI 15) to Jiquan (HT 1), from Binao (LI 14) to Jianyu (LI 15), from Jianqian (Extra) to Jianhou (Extra) and from Hegu (LI 4) to Houxi (SI 3) and so on. After qi arrival by lifting-thrusting and twirling-rotating manipulations, even reinforcing-reducing method was performed till patients had sore, heavy,

				distension sense in the entire shoulder and wrist, which might radiate to the forearm and fingers. Routine acupuncture method was performed for the adjunct acupoints, and even reinforcing-reducing manipulation was applied after qi arrival.<br>**CG**: CP the symptom-oriented treatments and the prevention of complications.
Zarnigar 2012	EGa: 45′ Massage with *Roghane Qust* alternative days for 2 months<br>EGb: Exercises 45′ alternative days for 2 months	Conventional unani medicine	Paretic upper and lower limbs and back on vertebrae	**EGa:** Dalak motadil (moderate massage) with moderate constant pressure for 45 mnts with *Roghane Qust* in the direction of lymphatic and venous drainage<br>**EGb:** Trunk symetry and sitting control exercises, rolling on both sides, sitting from supine position from both sides, Sit-to-Stand transfer, standing and transfer to both sides and gait positions.<br>**CG:** Conventional unani medicine using munzij (concoction) 15 gm twice a day for 15 days and mushil (purgation) therapy alternative days for 1 week followed by muqawwi aasab (nerve tonic) 6 gm twice a day for 5 weeks
Ji, 2013	30′Tuina massage + 20′balance training 1/day for 30 days	20′balance training for 30 days	All the body	**EG:Tuina massage:** From Dazhui (GV 14) to Changqiang (GV 1) along the pathway of the Du Mae (Governor Vessel, along the spine), the Bladder Meridian (along the sides of the spine) on the back and Jiaji (EX-B 2) points. Tuina manipulation: Gun Rolling manipulation (using the ulnar side of the hand as a kneader with a fist or handgun shape), An-Pressing, Ca-Rubbing, Rou-Kneading and Tanbo-Plucking. Prone position. relax the back muscles using Gun-Rolling manipulation 2-3 times from top to bottom. An-Press and Rou-Knead the acupoints from Dazhui (GV 14) to Changqiang (GV 1) along the Governor Vessel using the thumb or thenar eminence 3-5 times till a warm sensation occurs. Apply Gun-Rolling and An-Pressing manipulations to the Bladder Meridian on the back 5-6 times from top to bottom. Tanbo-Pluck the sacrospinous muscles on both sides using soft but penetrating manipulation. Follow this with heavy An-Pressing and Rou-Kneading Jiaji (EX-B 2) points. Ca-Rub the Governor Vessel and Bladder Meridian till a warm sensation appears.<br>**CG:** First, experience the feeling of standing with the help of a standing bed and correct the standing posture in front of a mirror. Next, exercise standing with and without support of the standing bed. Regarding weight-bearing exercise of the legs, first stand with help (by controlling the key areas in the knee and low back) or stand against a wall; try gradually to stand without help and exercise standing with separated legs, standing by placing two legs together and standing in a straight line; and then shift from standing with weight-bearing of both legs to standing on the affected leg. Ask the patient to lift arm and reach different objects in a standing posture. Exercise of back, front, left and right shift of the body weight in a balance trainer can improve the affected leg's weight-bearing and standing balance ability. After this, the patient can continue with head, upper limb, torso and lower limb movements in a standing posture and exercise standing in a balance board.
Li, 2012	Tuina massage + Electric acupuncture for 6 weeks	NDT + PNF for 6 weeks	Upper limb	**EG: Electric acupuncture**: Sanjian (LI 3), Houxi (SI 3), Zhongzhu (SJ 3), Jianzhongshu (SI 15), Jianliao (SJ 14), Jianyu (LI 15), Shousanli (LI 10), Waiguan (SJ 5) and Tianzong (SI 11). Operation: The patient lay on the healthy side. All acupoints were sterilized and pricked rapidly with filiform needles (No. 30) in the muscular layer from the distal to the proximal end of the affected limb, using the lifting, thrusting and twirling technique with the uniform reinforcing-reducing method to induce the needling sensation. Two of three pairs of acupoints - Sanjian and Jianyu, Houxi and Jianliao, or Zhongzhu and Jianzhongshu - were alternately selected for connection to Han's electric acupuncture apparatus (LH202H) with a current sufficient to make the muscles around the points tremble slightly and to cause slight pain to the patient. The needles were left in place for 25′. At the points that were not connected to the electric apparatus, the uniform reinforcing-reducing method was performed twice, for about 15′′ each time.<br>**EG:Tuina massage**: from forearm to shoulder: Manipulation of forearm (10′): The patient lay on the back or healthy side. The doctor used his/her palm and five fingers to gently press, rub and massage the patient's hand, wrist and elbow, especially at the Jing point, Shu point and He point of the Three Yang Channels, and then helped the patient to flex his/her fingers, palm and wrist without pain. Pressure could be applied at Chize, Shaohai, Shousanli and Hegu. Manipulation of shoulder (10′): Rolling was performed from the elbow to the shoulder, especially along the Three Yang Channel distribution area, gradually increasing the force at a frequency of 30 rolls per minute. During the procedure, Jianzhongshu, Jianlao and Jingyu could be pressed and rubbed as appropriate. Passive

				movement of shoulder joint (5′): The patient was in a sitting position. The doctor, with one hand holding the patient's elbow and the other hand holding the shoulder, gently helped the patient to move his/her shoulder joint in a gradually increasing range without pain. **CG:** Sitting position. The doctor, placing one hand on the affected pectoralis major muscle and the other hand on the lower angle of the scapula, helped the patient to move the scapula upward and downward, left and right for 15′ to relax the shoulder joint. 2) Pose training included maintaining a correct lying position, regularly changing the body position NDT exercise to clench the fist, changing from the prone to sitting position in bed, and changing from the sitting position to standing for 15′ PNF training was performed for 10′.
Zhang X, 2013	20′ Tuina massage + 60′CP 1/day for 5 days/week for 3 weeks	20′Acupuncture + 60′CP 1/day per 5 days/week for 3 weeks	Upper/lower limb	**EG: Tuina massage:** Apply the technique to the abdomen on the predominant side of the spasm (flexor side); gently hit the extensor muscles, rub the palms on the lower side (extension side) of the spasm, until the skin is warm; pressing the rub and pluck method on the upper limbs and shoulder Zhen, Quchi, Shousanli, Waiguan, Hegu and other acupuncture points; lower limb loop jump, Chengfu, Weizhong, Chengshan, Xiexi, Zusanli and other acupuncture points; aim and pinch the upper limbs Shao Shang, Shang Yang, Shao Ze, Shao Chong and other points. Lower limbs: Yinbai, Zhiyin, Lidui, Foot Qiaoyin and other points.<br>**CG: Acupuncture points** take the parietotemporal anterior oblique line and Baihui; body acupuncture points are mainly acupuncture points on the extensor side of the upper limb on the paralyzed side, such as Jianliu, Jianzhen, Quchi, Shousanli, Waiguan, Hegu, etc. Lower Extremities It mainly takes the lateral points of the flexor muscles, such as the loop jump, Chengfu, Fengshi, Yanglingquan, Xuanzhong and Xiexi.<br>**CP**: NDT and functional training for upper and lower limb: scrubbing, pushing heavy objects, panels, stacking paper boxes, cutting paper, picking up beans, stringing beads, throwing balls, screwing nuts, twisting wires, pinching play doug, dynamic sitting balance,sitting to stand, static cycling.
Zhang Y, 2013	30′ Tuina massage + 30′ acupuncture 1/day for 3 weeks	1 hour CP for 3 weeks	Paretic upper/lower limbs	**EG:** Eight acupoints on the affected side were selected for needling and massaging: Jianyu (LI15), Quchi (LI11), Hegu (LI4), Xuehai (SP10), Yanglingquan (GB34), Huantiao (GB30), Qiuxu (GB40), and Taichong (LR3).Massage treatment included point-pressing manipulations on the eight acupoints mentioned above and functional training of affected limbs. The point-pressing manipulations including pushing, pressing, and rolling, were operated with patients lying in bed and the functional training differed according to the patients' condition. Patients with limb atony received passive joint movements. Those with limb spasticity received passive joint movements, flexor inhibition, and extensor facilitation movements.<br>**CG:** CP: NDT. Therapeutic handling, facilitation, inhibition, and key points of control. Before being discharged from the hospital, patients had been clearly informed of a reasonable long-term rehabilitation plan and health education (Straus et al., 2002).
Thanakiatpinyo, 2014	60′ Thai massage alternative days for 6 weeks	CP	Paretic upper/lower limbs	**EG: Traditional Thai Massage** using only thumb and hand pressing at the point without traction or stretching. The basic massage lines were pressed for 10′ and the major signal points were pressed for 30′per point. Massage points included in this study were located in the leg, back, abdomen, arm, shoulder, and neck regions<br>**CG**: **CP:** Range of motion exercises, strengthening exercises for the paretic limbs, balance exercises for sitting and standing, and gait training.
Ahmed, 2015	*Munzij Balgham* once a day for 12 days + *Mushile Balgham* for 2 days + 15′massage 1/day for 2 weeks	Western medicine (drugs) for 4 weeks	Spine and paretic upper/lower limbs	**EG**: *Munzij Balgham* (Joshanda (decoction), Gulqand. On 13th day, the ingredients of Mushile Balgham were added to Munzij Balgham. On 15th day, massage was started using 20 ml of warm Roghan Malkangani.<br>**CP**: Piracetam, 800 mg, twice a day; Lisinopril, 2.5 mg, once a day; Aspirin 5 mg, once a day in tablet form.
Han, 2015	15′ Tuina massage 2/day + herbal medicine (oral) 15 g with warm water for	20′/day CP for 3 weeks	Paretic upper limb	**EG:** The Lung Meridian and the Large Intestine Meridian; the Pericardium Meridian and the Triple Energizer Meridian; the Heart Meridian and the Small Intestine Meridian. First, the practitioner applied heavy fast Gun-rolling and Na-grasping manipulations to the three yin meridians of Hand from the shoulder joint towards the wrist; then applied gentle slow Rou-kneading and Ca-rubbing manipulations to the three yang meridians along ulnar side of the arms (gut vessels: large intestine, triple energizer and small intestine) towards the shoulder joint. The practitioner was supposed to increase the force gradually until the patient felt soreness, numbness,

	each dose, 3 doses a day for 3 weeks			distension and mild pain. Forceful and even force were especially focused on the Five Shu-Transmitting points and Luo-Connecting points. Patients took spasticity-alleviating and meridianunblocking formula orally. Traditional phytotherapy, these plants are used for the mobilization of blood and energy, toning the kidney and the digestive system Ingredients: 10 g of Jiang Can (Bombyx Batryticatus), Quan Xie (Scorpion), Dang Gui (Radix Angelicae Sinensis) and Bai Shao (Radix Paeoniae Alba) respectively, Shui Zhi (Hirudo) 3 g, and 15 g of Huang Qi (Radix Astragali) and Sheng Di Huang (Radix Rehmanniae) respectively. Method: The above ingredients were ground into fine powder and stored in a bottle after filtration by a 100-mesh sieve. Patients took 15 g with warm water for each dose, 3 doses a day.<br>**CG**: anti-spasticity rehabilitation training. The training includes good limb position, maintaining of joint range of motion, alleviation of spasticity around body movement control points, static muscle stretching, and passive movement..
Di, 2017	15′ Tuina massage 2/day for 3 weeks	20′/day CP for 3 weeks	Paretic upper limb	**EG: Tuina massage**: The Lung Meridian and the Large Intestine Meridian; the Pericardium Meridian and the Triple Energizer Meridian; the Heart Meridian and the Small Intestine Meridian. First, the practitioner applied heavy fast Gun-rolling and Na-grasping manipulations to the three yin meridians (heart, lung and pericardium) which energetical movement is from body to upper extremity; then applied gentle slow Rou-kneading and Ca-rubbing manipulations to the three yang meridians of hand from the wrist towards the shoulder joint. The practitioner was supposed to increase the force gradually until the patient felt soreness, numbness, distension and mild pain. Forceful and even force were especially focused on the Five Shu-Transmitting points and Luo-Connecting points.<br>**CG:** The training included good limb position, maintaining of joint range of motion, alleviation of spasticity around body movement control points, static muscle stretching, and passive movement.
Lee, 2017	30′Swedish massage using essential oil + foot bath + CP 1/day per 5 days/week	30′CP 1/day per 5 days/week	Back and foot	**EG: Swedish massage:** effleurage, petrissage, friction, tapotement, and vibration with ten drops of juniper, seven drops of lavender, five drops of orange, three drops of patchouli, and eight drops of rosemary were used, and for back massage, essential oil was blended to 3% of the whole volume with 50 ml of jojoba oil the foot bath is defined as immersing the feet in water (40 °C) filled up to the ankle in the foot bath.<br>**CP:** NR
Yang, 2017	20–25′ per limb Tuina massage + 80′ CP a day per 5 days/week for 4 weeks	20–25′ per limb Sham Tuina massage + 80′ CP a day per 5 days/week for 4 weeks	Paretic upper/lower limbs	**EG:** Upper limb: The therapists applied their hands to roll on the anterior, lateral and posterior aspects of affected shoulder about 3′; The operating hand rolling and thumb waving pressing manipulation on the flextors of the arm from the shoulder to the wrist about 5′ The operating hand rolling and thumb waving pressing manipulation on the extensors of the arm from the shoulder to the wrist about 5′; Finger pressure on 8 acupuncture points: Jianyu (LI15), Jianliao (SJ14), Neiguan (PC6), Quchi (LI11), Shousanli (LI10), Waiguan (SJ50), Yangchi (SJ4), Hegu (LI4); Operating grasping manipulation on Jiquan (HT1) and the arm; Twisting the fingers; Operating pulling, rotating and shaking, traction, and stretching and flexing manipulation on the elbow, wrist and fingers; Rubbing the elbow and the wrist until patients felt warm; The whole procedure lasted about 20-25′. The patients should sense mild to moderate soreness and distension without pain. Lower limb: The therapists aplled their hands to roll on anterior, lateral and posterior hip of the affected side about 3 minutes; Operating hand rolling manipulation on the flextors of the lower limb from the hip to the ankle about 5 minutes; 3. Finger pressure on 6 acupuncture points: Huantiao (GB30), Yinmen (BL37), Weizhong (BL40), Chengshan (BL57), Sanjinjiao (SP6), Taixi (KI3); Operating grasping manipulation on posterior lower limb; Operating hand rolling and thumb waving pressing manipulation on the extensors of the lower limb from the hip to the ankle along the gallbladder and stomach meridian, about 5′; Finger pressure on 9 acupuncture points: Xuehai (SP10), Futu (ST32), Fengshi (GB31), Zusanli (ST36), Yanglingquan (GB34), Tiaokou (ST38), Jiexi (ST41), Taichong (LR3), Neiting (ST44); 6. Operating pulling, rotating and shaking, traction, and stretching and flexing manipulation on the hip, knee, and ankle; Operating grasping manipulation on anterior and lateral lower limb; Rubbing the knee and ankle until patients sensed warm; The whole procedure lasted about 20-25′. The patients should feel mild to moderate soreness and distension without pain.<br>**CG:** Sham Tuina is gentle rubbing session. Participants should feel nothing but gentle moving. The movements appeared as the real Tui Na therapy so that the participants could not distinguish it from the real Tui Na.<br>**CP**: positioning to reduce spasticity; muscle stretching; neuro-muscular facilitation technique therapy; electrical stimulation.
Wang, 2019	40′ Tuina massage + 2	2 hours CP 30′ OT	Paretic upper limb	**EG: Tuina massage**:Rolling on the shoulder of the paralyzed side for approximately 3′; through hand rolling and thumb waving pressing, the arm flexors of the paralyzed limbs were manipulated from the shoulder to the wrist for approximately 5′; through hand

	hours CP + 3’OT per 5/week for 4 weeks			rolling and thumb waving pressing, the arm extensors of paralyzed limbs were manipulated from the shoulder to the wrist for approximately 5’; finger pressure was applied on acupuncture points in the paralyzed limbs, including two points on the shoulder [Jianyu (LI15) and Jianliao (SJ14)], Quchi (LI11) on the lateral end of the elbow crease, three points on the forearm [Neiguan (PC6), Waiguan (SJ50), Shousanli (LI10)], and two points on the hand [Yangchi (SJ4), Hegu (LI4)]. Moreover, the Jiquan (HT1) acupoint on the paralyzed arm was manipulated (Yang 2016) by grasping; the fingers were manipulated by twisting; the elbow, wrist and fingers were manipulated by pulling, rotating, shaking, stretching and flexing; and the elbow and wrist were manipulated by rubbing<br>**CG:** Muscle stretching, proper positioning and exercises to reduce spasticity, neuromuscular facilitation techniques therapy, and antagonistic muscle electrical stimulation therapy, 2 h per time; and (2) a 30’ of OT training according to the patients’ individual conditions to carry out activities of daily living (ADL), including manual simulation operation, sports and entertainment training.
Xie, 2019	45’ Tuina massage + 45’CP 1 session/day (10 days as a course), at a 2-day interval, for 3 courses.	45’CP 1 sesion/day (10 days as a course) at a 2-day interval, for 3 courses.	Paretic lower limb	**EG: Tuina massage**: one-thumb Tuipushing, Gun-rolling, Rou-kneading, An-pressing and Tanbo-plucking. The patient first took a supine position. The physician first applied one-thumb Tui-pushing to Guanyuan (CV 4) and Qihai (CV 6), and then applied the rolling method to the anterior and lateral flank of the patient's lower limb from the anterior superior iliac spine to the back of the foot, especially focusing on acupoints such as Futu (ST 32), Zusanli (ST 36) and Jiexi (ST 41), with passive flexion and extension exercise of the affected limb joint, for about 25 min each time. The patients then took a prone position. The doctor first An-pressed and Rou-kneaded the patient’s back, buttocks and the back of the thigh, focusing on acupoints such as Xinshu (BL 15), Pishu (BL 20), Weishu (BL 21), Geshu (BL 17), Ganshu (BL 18), Danshu (BL 19), Shenshu (BL 23), Huantiao (GB 30), Weizhong (BL 40) and Kunlun (BL 60), for about 10 min. Finally, the patient lied on the healthy side. The doctor applied Gun-rolling manipulation to the outside of the affected limb in combination with the passive movement of the hip and knee joint for about 10’<br>CG: CP: The treatments included range of motion (ROM) training, activities of daily living training, NDT, gait training, physical therapy and occupational therapy. The patients were prescribed with lower limb orthosis training when necessary.
Zhang, 2020	30-45’ Tuina massage + 20’ electroacupuncture + 60’CP 1/day for 40 days	30-45’ Tuina massage + 20’ electroacupuncture 1/day for 40 days	Paretic upper/lower limbs	**Acupuncture:**The patients’ limb acupuncture in motor area, the upper limb subjected to Quchi, Handsanli, Waiguan, Daling and Hegu and the lower limb subjected to ring jump, hanging bell, Sanyinjiao, Taixi, Zusanli and Yanglingquan. After needling according to regular acupuncture method, acupuncture therapeutic apparatus was connected.<br>**Tuina massage:** Pressing, pinching, pushing, holding.<br>**CP:** weight-bearing training in anti.spasm mode, back.body training with the heels touching tne ground.Family members assisted in training, 2 times a day, 10-15’ each time during 20 days a cours and the 2º course was started 3 days after 1st course.

CG: control group, CP: conventional physiotherapy, EG: experimental group, NDT: Neurodevelopmental treatment, NR: Not reported, OT: occupational therapy, PNF: proprioceptive neuromuscular facilitation, (’):minutes

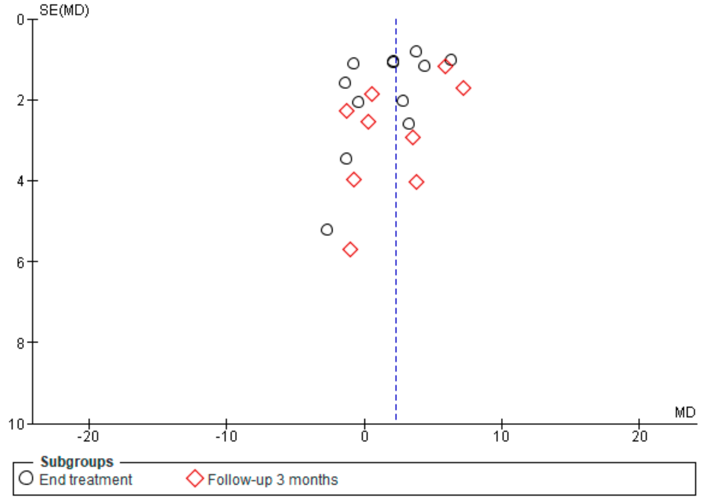

Supplemental Figure S1: Funnel plot of comparison Tuina massage plus conventional physiotherapy (CP) versus CP for motor-function evaluated by Fugl Meyer Assessment Scale

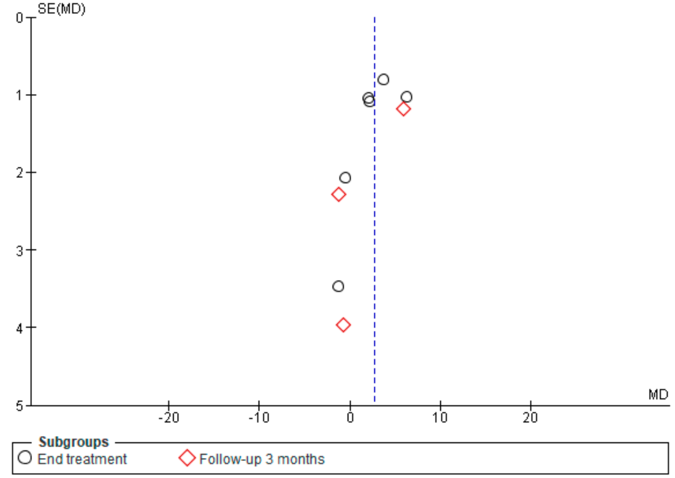

Supplemental Figure S2: Funnel plot of comparison Tuina massage plus conventional physiotherapy (CP) versus CP for upper and lower limb motor function in subacute stage (≤3 months) evaluated by Fugl Meyer Assessment

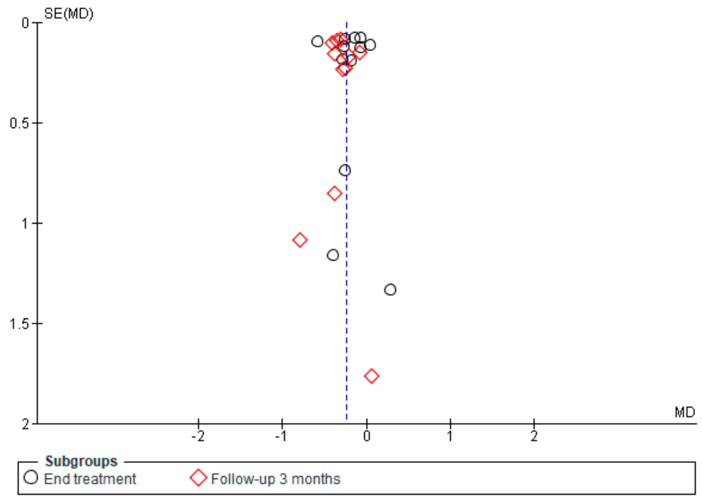

Supplemental Figure S3: Funnel plot of comparison Tuina massage plus conventional physiotherapy (CP) versus CP for spasticity evaluated by modified Ashworth Scale

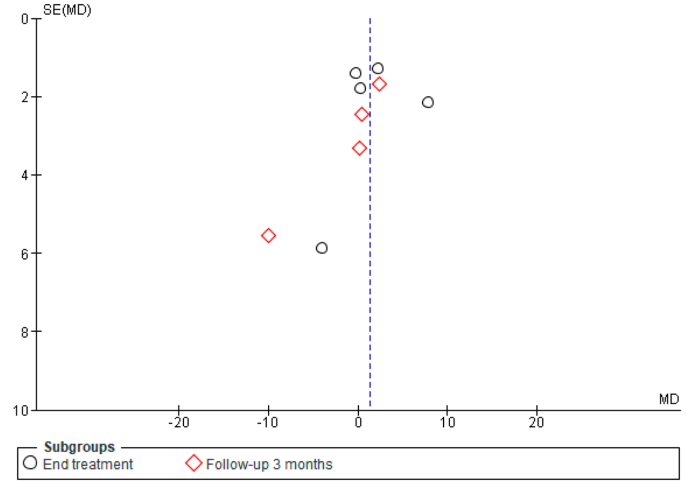

Supplemental Figure S4: Funel plot of Tuina massage plus acupuncture versus conventional physiotherapy for motor-function evaluated by Fugl Meyer Assessment Scale

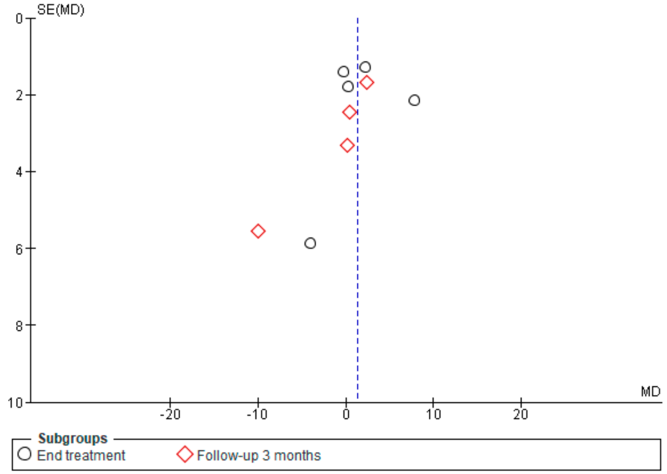

Supplemental Figure S5: Funnel plot of comparison Tuina massage plus conventional physiotherapy (CP) versus CP for activities of daily living assessed by modified Barthel Index
